# Optimization of an Ultrasound-Assisted Extraction Technique and the Effectiveness of the Sunscreen Components Isolated from *Bletilla striata*

**DOI:** 10.3390/molecules29122786

**Published:** 2024-06-12

**Authors:** Yan Luo, Zhenyuan Tan, Hancui Zhang, Shuai Tang, Suren R. Sooranna, Jizhao Xie

**Affiliations:** 1 Life Sciences Institute, Guangxi Medical University, Nanning 530021, China; luoyan@gxmu.edu.cn; 2 Guangxi Key Laboratory of Regenerative Medicine, Guangxi Medical University, Nanning 530021, China; 3 Department of Pharmacology, Guangxi Medical University, Nanning 530021, China; 370071@sr.gxmu.edu.cn (Z.T.); zhanghancui@sr.gxmu.edu.cn (H.Z.); gcp4279629@126.com (S.T.); 4 Department of Metabolism, Digestion and Reproduction, Imperial College London, Chelsea and Westminster Hospital, London SW10 9NH, UK; s.sooranna@imperial.ac.uk; 5 Life Science and Clinical Research Center, Youjiang Medical University for Nationalities, Baise 533000, China; 6 NMPA Key Laboratory for Quality Monitoring and Evaluation of Traditional Chinese Medicine, Nanning 530021, China

**Keywords:** *Bletilla striata*, sunscreen components, orthogonal experiments, ultrasound-assisted extraction

## Abstract

*Bletilla striata* is the dried tuber of *B. striata* (Thund.) Reichb.f., which has antibacterial, anti-inflammatory, anti-tumor, antioxidant and wound healing effects. Traditionally, it has been used for hemostasis therapy, as well as to treat sores, swelling and chapped skin. In this study, we used the ultraviolet (UV) absorbance rate of *B. striata* extracts as the index, and the extraction was varied with respect to the solid–liquid ratio, ethanol concentration, ultrasonic time and temperature in order to optimize the extraction process for its sunscreen components. The main compounds in the sunscreen ingredients of Baiji (*B. striata*) were analyzed using ultra-high-performance liquid chromatography combined with quadrupole time-of-flight tandem mass spectrometry. The sunscreen properties were subsequently evaluated in vitro using the 3M tape method. The results show that the optimal extraction conditions for the sunscreen components of *B. striata* were a solid–liquid ratio of 1:40 (g/mL), an ethanol concentration of 50%, an ultrasonic time of 50 min and a temperature of 60 °C. A power of 100 W and an ultrasonic frequency of 40 Hz were used throughout the experiments. Under these optimized conditions, the UV absorption rate of the isolated sunscreen components in the UVB region reached 84.38%, and the RSD was 0.11%. Eighteen compounds were identified, including eleven 2-isobutyl malic acid glucose oxybenzyl esters, four phenanthrenes, two bibenzyl and one α-isobutylmalic acid. An evaluation of the sunscreen properties showed that the average UVB absorption values for the sunscreen samples from different batches of *B. striata* ranged from 0.727 to 1.201. The sunscreen ingredients of the extracts from *B. striata* had a good UV absorption capacity in the UVB area, and they were effective in their sunscreen effects under medium-intensity sunlight. Therefore, this study will be an experimental reference for the extraction of sunscreen ingredients from the *B. striata* plant, and it provides evidence for the future development of *B. striata* as a candidate cosmetic raw material with UVB protection properties.

## 1. Introduction

Studies suggest that excessive ultraviolet (UV) radiation can cause skin redness, tanning, sunburn and even the risk of skin cancer [1,2]. Sunscreen preparations can effectively prevent overexposure to UV radiation and significantly reduce the skin damage caused [3,4]. However, the properties of the sunscreens currently in use have certain shortcomings, including poor skin sensation with some their physical effects, as well as irritation and allergic reactions caused by the chemical components in the skin [5]. Compared with some of these physical and chemical sunscreen methods, natural plant sunscreen constituents have the advantages of high safety, and they can be more comfortable when applied to the skin. Therefore, in recent years, some substances for performing this task have been obtained from Chinese herbal medicines and have increased in popularity, and this is one of the major directions of research and development for sunscreen skincare products [6].

*Bletilla striata* (Thund., named ‘‘Bai Ji” in Chinese) is the dried tuber of *B. striata* (Thund.) Reichb.f., which is a relatively rare traditional Chinese medicine (TCM), with a history of over 1500 years [7]. The *B. striata* plant is mainly distributed in the humid river valleys and coastlands in the most southeastern three provinces of China. Outside China, its global distribution is limited, only growing in the Korean Peninsula, Japan and Myanmar [8]. Traditionally, it has been used for hemostasis therapy, as well as to treat sores, swelling and chapped skin [9]. Additionally, in ancient times, the dry tubers of *B. striata* were ground into a powder and then applied to the skin in order to achieve beautifying and skincare effects. Nowadays, some regions of China still retain this ancient method of skincare [10]. The main chemical components of *B. striata* consist of a variety of chemical substances, such as polysaccharides, benzenes, dihydrophenanthrolines and phenanthrenes, flavonoids, diphenylenes and diphenylene ethers and quinones [8,11]. Therefore, *B. striata* has a wide range of bio-pharmacological activities, such as bacteriostasis, anti-inflammatory [12], hemostasis [13], anti-tumor [14], cell-growth-promoting and wound healing properties [11,15]. Additionally, it has been shown to have a significant antioxidant effect in vitro [8,16,17]. Most of the chemical structures of *B. striata* compounds contain benzene ring skeletons and even multiple phenolic hydroxyl groups. It has been reported that polyphenols have good free radical scavenging and antioxidant properties [18,19], and these are capable of absorbing UV light and inhibiting the skin aging caused by this type of radiation [20]. As a TCM used for whitening the skin [21], it was speculated that *B. striata* contained components that had sunscreen properties. At present, the extraction methods for the total polyphenols in *B. striata* include the traditional heated reflux [22] and flash extraction methods [23], as well as the high-shear mixing emulsification technique [24]. These conventional methods have been in use for many decades, but they have major limitations. The flash extraction method and the high-shear mixing emulsification technique require specialized instrumentation which are not common in laboratories. Therefore, a practical and efficient extraction method is crucial for its wider use. In this context, the application of an ultrasound technique to the extraction of bioactive components is considered as a promising alternative, and it is emerging as a green technology due to the lower costs during operation. It has the advantages of shorter operation times and lower energy consumption when compared to conventional methods [25].

However, at present, there are few studies on the optimization process for the ultrasound-assisted extraction of polyphenols from *B. striata.* There are many studies on the antioxidant activity of polyphenols from *B. striata* but only a few reports on the sunscreen properties of extracts from *B. striata*. In order to explore the sunscreen components, the extraction process and the sunscreen effects of *B. striata*, we used an ultrasound-assisted method to extract its sunscreen compounds. The UV absorption rate was used as an index to optimize the conditions of the extraction process for the sunscreen ingredients obtained from this plant. Four parameters, including the solid–liquid ratio, ethanol concentration, ultrasonic time and temperature, were varied. The main compounds in the sunscreen ingredients of Baiji (*B. striata*) extracted under the optimal processing conditions were then identified using ultra-high-performance liquid chromatography combined with quadrupole time-of-flight tandem mass spectrometry (UPLC-Q-TOF-MS). The 3M tape method was subsequently used to preliminarily evaluate the sunscreen effects of the ingredients obtained with a view to providing a reference for the extraction procedure. This will aid in the future development and utilization of *B. striata* extracts as a functional ingredient in the skincare industry.

## 2. Results and Discussion

### 2.1. Screening of Sunscreen Components Extraction Conditions

#### 2.1.1. The Effect of Ultrasonic Time on the Extraction Effect of Sunscreen Ingredients from *B. striata*

**Figure 1:**
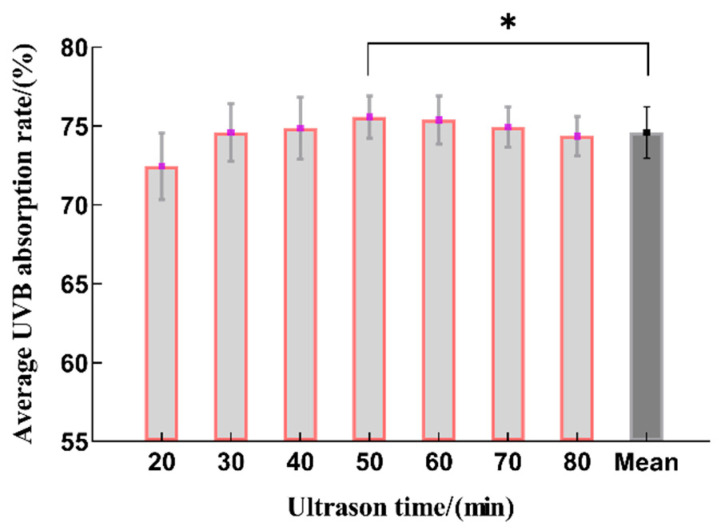
The effect on the sunscreen activity for different time periods used for extraction (*n* = 3, * represents *p* < 0.05).

As shown in Figure 1, it can be seen that within 20~50 min, the UVB absorption rate showed a trend of first increasing and then decreasing with the extension of the extraction time, and the extraction rate was the highest at 50 min, which is statistically significant when compared to the average value. This may be due to the prolongation of the time in the early stage and the continuous penetration of the extraction solution, resulting in an increase in the extraction rate. However, as the extraction time increased, the oxidation of polyphenols in the extraction solution accelerated, resulting in decreased yields [23]. Therefore, the optimal range for the ultrasonic extraction time was determined to be 40~60 min.

#### 2.1.2. The Influence of Ethanol Concentration on the Extraction Effect of Sunscreen Ingredients from *B. striata*

**Figure 2:**
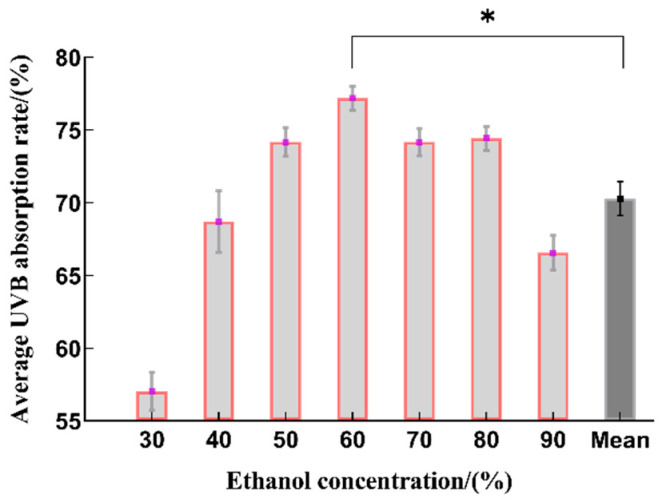
The effect on the sunscreen activity obtained by using different concentrations of ethanol for extraction (*n* = 3, * represents *p* < 0.05).

The UVB absorption rate showed a trend of first increasing and then decreasing as the ethanol concentration increased, with the highest extraction rate seen at 60% ethanol, which is statistically significant when compared to the average value (Figure 2). It is possible that as the ethanol volume fraction increased, the dissolution of some alcohol-soluble impurities and highly lipophilic components increased. These components affected the dissolution of polyphenolic compounds from the raw material particles, resulting in a decrease in the total phenol yield of the extract [23]. Therefore, the optimal range for the ethanol concentration was determined to be 50~70% ethanol.

#### 2.1.3. The Effect of the Solid–Liquid Ratio on the Extraction of Sunscreen Ingredients from *B. striata*

**Figure 3:**
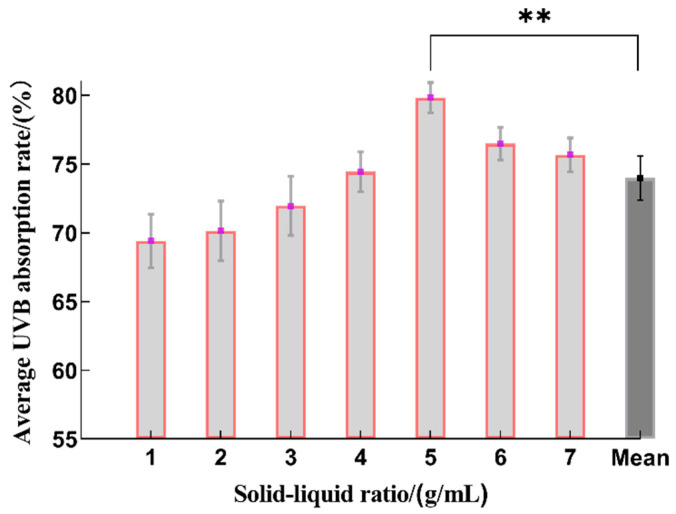
The effect on the sunscreen activity of different solid–liquid ratios during the extraction process. Ratios of solid–liquid of 1:10, 1:15, 1:20, 1:30, 1:40, 1:50 and 1:60 (g/mL) correspond to 1, 2, 3, 4, 5, 6 and 7 along the x-axis (*n* = 3, ** represents *p* < 0.01).

With respect to solid–liquid ratios from 1:10 to 1:60 (g/mL), the extraction rate of the sunscreen components increased with an increase in the matter solid–liquid ratio, and this reached its maximum at 1:40 (g/mL), which is statistically significant when compared to the average value (Figure 3). However, as the dose of the solvent increased, there was a relatively small decrease in the UVB absorption rate. This is probably because a greater percentage of the sunscreen components was released as the solvents increased when the solid–liquid ratio was less than 1:40 (g/mL). However, excessive use of solvents was not conducive to the extraction of the sunscreen ingredients because the solute became saturated in the solvent, which adversely affected the mass transfer efficiency [26]. Therefore, the optimal range for the solid–liquid ratio was determined to be 1:30~1:50 (g/mL).

#### 2.1.4. The Effect of Ultrasonic Temperature on the Extraction of Sunscreen Ingredients from *B. striata*

**Figure 4:**
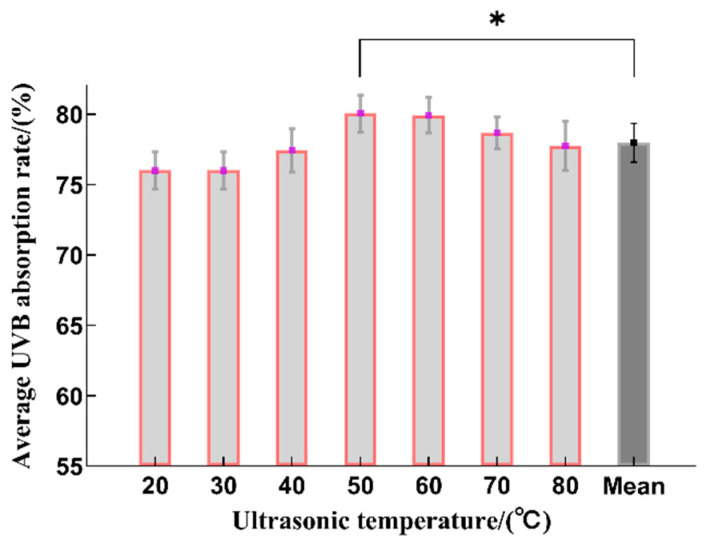
The effect on the sunscreen activity after using different ultrasonic temperatures during the extraction process (*n* = 3, * represents *p* < 0.05).

From 20 to 50 °C, the UVB absorption rate increased as the ultrasonic temperature rose (Figure 4). A plausible reason is that an increase in temperature can improve the mass transfer efficiency and facilitate the extraction of sunscreen substances [8]. The sunscreen substances had a maximum extraction rate at 50 °C, which is statistically significant when compared to the average value. At higher temperatures, the UVB absorption rate fell as the ultrasonic temperature rose. This may have been due to the degradation of the sunscreen components at the higher temperatures, thereby reducing the extraction yield. Therefore, the optimal range for the extraction temperature was determined to be 40~60 °C.

### 2.2. Statistical Analysis of the Orthogonal Test

According to the results of the single-parameter tests, the UV absorption of *B. striata* extracts in the UVA region was found to be relatively weak, with an average absorption rate of less than 30%. However, the UV absorption in the UVB region was relatively strong, with an average absorption rate of more than 55%. On the basis of the single-factor tests, the UVB absorption rate was used as the response value, with the solid–liquid ratio (A), the ultrasonic temperature (B), the ethanol concentration (C) and the ultrasonic time (D) as the self-variables. The above factors and levels were then transferred into an L9 (3^4^) orthogonal table (Table 1). The last column in the table refers to the UVB absorption rate, which is the experimental detection index. Each row in the table represents a set of parameter combination experiments, and all four columns are balanced, orthogonal and independent. A total of nine groups were used to conduct the analysis with the different combinations of processing parameters.

Range analysis can be used to analyze orthogonal experimental data, and it can demonstrate the advantages of factors, as well as the advantages and disadvantages of factors at specific levels. From the range analysis shown in Table 2, it can be seen that the R values (factor range value) obtained reflect a comparison of the results of the four parameters used for the extraction of sunscreen products from *B. striata*. The largest value is R_C_, followed by R_B_ and then R_A_, and R_D_ is the smallest. This indicates that the factors which affected the extraction of the sunscreen ingredients most, in order, were ethanol concentration, which had the greatest impact, followed by ultrasonic temperature, then the solid–liquid ratio and finally ultrasonic time had the least impact. When specifically combined with the optimal levels for each factor, it can be seen that the second level of factor 1 was the solid–liquid ratio, which was optimal at 1:40 (g/mL), and the third level of factor 2 was the ultrasonic temperature, which was optimal at 60 °C. In addition, the first level, which was the ethanol concentration of factor 3 (50%), and the second level of factor 4, which was the ultrasound time (50 min), were optimal. In summary, the optimal factor was found to be ethanol concentration. The optimal combination is A2B3C1D2. That is, a ratio of solid–liquid of 1:40 (g/mL), an ultrasonic temperature of 60 °C, an ethanol concentration of 50% and an ultrasonic time of 50 min. 

### 2.3. Verification Test

Three parallel verification tests were conducted based on the obtained optimized extraction process conditions. In Table 3, using a solid–liquid ratio of 1:40 (g/mL), an ultrasonic temperature of 60 °C, an ethanol concentration of 50% and an ultrasonic time of 50 minutes as the extraction conditions, the average UVB absorption rate of the extracted products from *B. striata* reached 84.38%. The average UVB absorption rate under these conditions was equivalent to the test results of No. 6 in Table 1, with an RSD of 0.11%. The results of this experimental group showed that the optimal extraction process conditions obtained through orthogonal experiments had the best effect.

### 2.4. Analysis Using UPLC-Q-TOF-MS 

**Figure 5:**
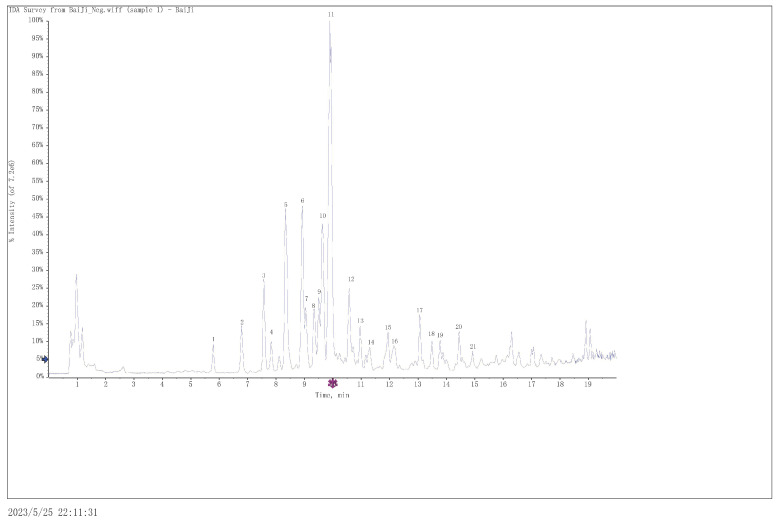
The total ion chromatogram obtained in the negative mode.

Using the abovementioned optimal extraction process conditions, the sunscreen substances in *B. striata* were extracted, and the compounds in the extracts were analyzed and identified using UPLC-Q-TOF-MS. Figure 5 represents a total ion chromatogram in the negative state obtained using optimized chromatographic and mass spectrometric conditions. Eighteen compounds were identified through analysis, and these included eleven 2-isobutyl malic acid glucose oxybenzyl esters, four phenanthrenes, two bibenzyl and one α-isobutylmalic acid (Table 4). The structures of these compounds contain benzene ring skeletons, and some also had phenolic hydroxyl groups. These groups give the compounds the ability to absorb UV radiation [27]. Therefore, analysis of the chemical components of the extracts showed that the extracts from *B. striata* had the effect of absorbing UV rays, and polyphenols are the components responsible for the sun-protective effects.

### 2.5. Evaluation of Sunscreen Effects in the UVB Region

In Table 5, the average absorbance values, Ai, of sunscreen Samples 1 and 2 in the UVB region as measured using the 3M tape method were 0.727 and 1.201, respectively. According to the sunscreen effect evaluation criteria shown in Table 6, if the absorbance was in the range of 0.5~1.0, this had a minimal UV protection effect, and the sample could be used in winter sunlight and summer morning and evening sunlight, as well as on cloudy days. If the absorbance was within the range of 1.1~1.5, this meant the sample had a medium UV protection effect, and it could be used in medium-intensity sunlight. It can be seen from the experimental results that the tested sunscreen samples had some sunscreening capacity. Therefore, Sample 1 could be used for basic sun protection in winter sun and on cloudy days, and Sample 2 could be used to protect against moderate sunlight.

The evaluation techniques to test the effectiveness of sunscreen products are divided into in vivo and in vitro methods. The in vivo method is based on the degree of the response of the human body to the production of erythema and pigmentation in the skin as caused by UV stimulation. Test results from the human body method are highly reliable, but there are also some obvious shortcomings. For example, testers need professional training, the test costs are high and large doses of UV rays can cause damage to the skin of the testers. Therefore, during the process of scientific research and product development, it is not practical to directly use in vivo methods to evaluate the sunscreen effects of samples. With the in vitro method, quartz sheets are often used to mimic biological tissues such as the cuticle and epidermis of the human body, UV-protective products are applied to these and the absorbance (transmittance) of the samples are measured using a UV spectrophotometer. The sun protection properties of a product can be evaluated based on the absorbance values obtained. Therefore, as in this study, it is more practical to use in vitro methods to evaluate the sun protection efficacy of UV-protective products during research and development [26].

**Table 4:** The individual components isolated from the ethanol extract samples of *Bletilla striata* after analysis by UPLC-Q-TOF-MS.

No.	t_R_ (min)	Extracting ions	Calculated (*m*/*z*)	Observed (*m*/*z*)	Error (ppm)	Area (%)	Formula	Main Secondary Fragment Ions	Identity	Category	References
1	5.787	[M − H]^−^	351.1297	351.1308	3.2	0.792	C_14_H_24_O_10_	171.0666, 127.0771	Dactylorhin C	2-isobutyl malic acid glucose oxybenzyl esters	[28,29]
2	6.788	[M − H]^−^	189.0768	189.0777	4.5	1.763	C_8_H_14_O_5_	171.0648, 129.0556	α-isobutylmalic acid	α-isobutylmalic acid	[28]
3	7.57	[M − H]^−^	619.2244	619.2259	2.5	3.261	C_27_H_40_O_16_	439.1605, 171.0661, 153.0554	Dactylorhin E	2-isobutyl malic acid glucose oxybenzyl esters	[28,29]
4	7.833	[M − H]^−^	593.1876	593.1881	0.9	1.039	C_28_H_34_O_14_	431.1346, 325.0930, 269.0802, 163.0399	2,4-dimethoxyphenanthrene-3,7-O-β-D-diglucopyranoside	Phenanthrenes	[28]
5	8.345	[M − H]^−^	887.319	887.3216	2.9	7.538	C_40_H_56_O_22_	619.2248, 439.1607, 179.0564, 171.0658, 153.0555	Dactylorhin A	2-isobutyl malic acid glucose oxybenzyl esters	[28,29]
6	8.925	[M − H]^−^	457.1715	457.1721	1.2	6.611	C_21_H_30_O_11_	171.0660, 161.0450, 153.0555, 129.0562, 127.0768, 123.0456	Gymnoside II	2-isobutyl malic acid glucose oxybenzyl esters	[29,30]
7	9.044	[M − H]^−^	723.5053	723.5049	−0.5	2.332	C_41_H_72_O_10_	661.2368, 481.1685, 439.1621, 153.0562	Unknown		
8	9.341	[M − H]^−^	661.2349	661.2369	3	2.593	C_29_H_42_O_17_	439.1610, 171.0663, 153.0557	Isomers	2-isobutyl malic acid glucose oxybenzyl esters	[30]
9	9.51	[M − H]^−^	853.5776	853.579	1.6	2.51	C_58_H_78_O_5_	661.2358, 481.1727, 439.1611, 171.0667, 153.0553	Unknown		
10	9.637	[M − H]^−^	929.3296	929.3323	2.9	6.621	C_42_H_58_O_23_	661.2353, 439.1610, 221.0662, 171.0666, 161.0443, 153.0555	Gymnoside Ⅲ	2-isobutyl malic acid glucose oxybenzyl esters	[28,29]
11	9.908	[M − H]^−^	725.2662	725.2686	3.3	19.932	C_34_H_46_O_17_	457.1726, 285.0984, 171.0662, 153.0555, 129.0561, 127.0769	Militarine	2-isobutyl malic acid glucose oxybenzyl esters	[28]
12	10.574	[M − H]^−^	971.3402	971.3432	3.1	3.396	C_44_H_60_O_24_	703.2466, 481.1735, 439.1619, 153.0554	Gymnoside VIII	2-isobutyl malic acid glucose oxybenzyl esters	[28,29,30]
13	10.963	[M − H]^−^	971.3402	971.3429	2.8	1.369	C_44_H_60_O_24_	749.2674, 703.2490, 661.2341, 481.1725, 439.1619, 153.0550	Habenarioside	2-isobutyl malic acid glucose oxybenzyl esters	[28,29]
14	11.287	[M − H]^−^	753.2611	753.2624	1.7	1.007	C_35_H_46_O_18_	439.1585, 171.0661, 153.0537	Unknown		
15	11.938	[M − H]^−^	241.087	241.087	0.8	2.088	C_15_H_14_O_3_	226.0640, 225.0566, 198.0686, 197.0600, 181.0678	Coelonin	Phenanthrenes	[31]
16	12.148	[M − H]^−^	1017.3609	1017.3621	1.9	1.67	C_49_H_62_O_23_	897.3407, 767.2793, 499.1825, 457.1680, 439.1532, 285.0985, 153.0559	Gymnoside IV	2-isobutyl malic acid glucose oxybenzyl esters	[28,29]
17	13.064	[M − H]^−^	1059.3715	1059.3738	2.2	2.24	C_51_H_64_O_24_	837.3036, 791.2795, 439.1623, 569.2024, 661.2389, 153.0557	Gymnoside X	2-isobutyl malic acid glucose oxybenzyl esters	[28,29]
18	13.49	[M − H]^−^	243.1027	243.1034	3.8	1.017	C_15_H_16_O_3_	243.1044, 227.0722, 183.0819, 136.0539, 106.0424	Batatasin Ⅲ	Bibenzyl	[31]
19	13.775	[M − H]^−^	347.1289	347.1296	2.1	0.912	C_22_H_20_O_4_	332.1050, 331.0973, 304.1091, 239.0707, 238.0632, 237.0562, 225.0550	1-(p-hydroxybenzyl)-4-methoxy-9,10-dihydroph -enanthrene-2,7-diol.	Phenanthrenes	[32]
20	14.446	[M − H]^−^	481.1657	481.1660	0.7	1.755	C_30_H_26_O_6_	466.1434, 465.1347, 451.1219, 435.1243, 225.0568	Blestrianol A or Gymconopin C or Blesriarene A	Phenanthrenes	[32]
21	14.921	[M − H]^−^	455.1816	455.1866	0.4	0.601	C_29_H_28_O_5_	361.1440, 346.1183, 331.0959, 255.1010	3,3’-dihydroxy-2’,6’-bis(p-hydroxybenzyl)-5-methoxybibenzyl	Bibenzyl	[33]

There are certain limitations to this study. Firstly, the extract from *B. strata* is a dark and unattractive product due to the presence of pigment. For its use as a cosmetic raw material, the pigment must be removed, which will improve its appearance and presence. Therefore, decolorization treatments will need to be considered in the future. Secondly, the extract of *B. strata* has to be evaluated for its toxicity in order to be used on human skin. Therefore, the extract requires local toxicological safety evaluation, such as multiple skin irritation, acute eye irritation, skin allergy and further tests [34]. Thirdly, as of yet, there is a lack of availability of pure compounds for use as internal standards, and this will have to be remedied in future experiments. In future studies, these limitations must be addressed prior to its use as a product in the cosmetic industry.

**Table 5:** The UVB absorption values obtained for the sunscreen components of different fractions.

No.	Wavelength (nm)					
	A_280_	A_290_	A_300_	A_310_	A_320_	A_i_
Control	0.537	0.570	0.468	0.336	0.203	0.409
1	0.948	0.873	0.790	0.622	0.403	0.727
2	1.569	1.350	1.221	1.054	0.811	1.201

**Table 6:** Evaluation of the sunscreen effects of *B. striata*.

Absorbance (A)	Sunscreen Effect	Conditions of Use
0.5~1.0	Minimum UV protection effect	Winter sunshine, summer morning and evening sunshine and overcast days
1.1~1.5	Moderate UV protection	Moderate sunlight
1.6~2.0	High-efficiency protection against ultraviolet radiation	Outdoor work, strong sunlight in summer
2.1	Fully protected against UV radiation	Outdoor work, strong sunlight in summer

## 3. Materials and Methods

### 3.1. Materials and Instruments

#### 3.1.1. Materials and Chemical Regents

*B. striata* (batch number: 20181101) was purchased from Guangdong Huiqun Herbal Medicine Co., Ltd. (Shantou, China). HPLC-grade acetonitrile and formic acid were from Merck (Merck Group, Darmstadt, Germany). Stearic acid, octadecyl alcohol, Tween 80, Span 60, liquid paraffin, vitamin E, carbomer, EDTA disodium, polydimethylsiloxane and glycerin were from Shanghai Yuanye Biotechnology Co., Ltd. (Shanghai, China). Sodium sorbate was from Henan Heisenlin Biotechnology Co., Ltd. (shangqiu, China), and medical tape was purchased from 3M Company (St. Paul, MN, USA). All the other reagents used were analytical-grade.

#### 3.1.2. Instruments

The UPLC-Q-TOF-MS analyses were performed on an AB Sciex Triple 6600+ mass spectrometer (AB Sciex Pte. Ltd., Atlanta, GA, USA). A WJX-A250 high-speed multi-function crusher (Shanghai Yuanwo Industry and Trade Co., Ltd., Shanghai, China) and a KQ-500DE CNC ultrasonic cleaner (Kunshan Ultrasonic Instrument Co., Ltd., Kunshan, China) were used throughout this study. In addition, a UV-mini 1240 UV spectrophotometer (Shimadzu Corporation, Kyoto, Japan), A TD5A-WS medical centrifuge (Xiangyi Centrifuge Instrument Co., Ltd., Changsha, China) and an XS205DU One Hundred Thousand Electronic Balance (Mettler Toledo Co., Ltd., Zurich, Switzerland) were also used.

### 3.2. Methods

#### 3.2.1. Extraction of the Sunscreen Ingredients from *B. striata*

The herbs of *B. striata* were first crushed in a grinder and sieved through a 100-mesh sieve. Then, 1.0 g of *B. striata* powder was placed in a 100 mL triangular flask with a stopper and subjected to ultrasonic extraction at 100 W and at an ultrasonic frequency of 40 Hz. Different ultrasonic times, ethanol concentrations, solid–liquid ratios and ultrasonic temperatures were used. The extracted products were kept at room temperature, centrifuged and filtered, the filtrates were transferred into a 100 mL volumetric flask and water was added to obtain a standardized volume.

#### 3.2.2. The Determination of the Absorbance UV

UV can generally be divided into three wave bands, namely UVA (320~400 nm), UVB (280~320 nm) and UVC (200~280 nm) in the long-, medium- and short-wave zones, respectively [35]. The transmission of UVC in the short-wave zone can only reach the cuticle, and most of it is blocked by ozone before reaching the ground. It is generally believed that UVC in the short-wave zone will not cause damage to the skin [36].

A total of 25.0 mL of the test solution obtained in Section 3.2.1 was transferred into a 50 mL volumetric flask, and this was used as the blank control to measure the UV absorbance values of the extracts of *B. striata* in the UVB and UVA regions, respectively. This was then used to evaluate the UV absorption (sun protection) capacity of the extracts. The values obtained were converted into the UV absorbance values. These were used to calculate the UV absorbance according to the formula [37]:

UV absorbance/% = 100% − average transmittance T

#### 3.2.3. Single-Factor Tests on the Extraction Process of Sun Protection Ingredients from *B. striata*

Briefly, 7 × 1.0 g of powder of *B. striata* was weighed, and the ultrasonic temperature used was 50 °C, the ratio of material to liquid was 1:20 (g/mL) and the ethanol concentration was 50%. These were extracted by ultrasonication for 20, 30, 40, 50, 60, 70 and 80 minutes, respectively. The extracts were brought to room temperature and filtered, and the filtrates were made up to 100 mL in volumetric flasks. Similar extractions were carried out to determine the concentration of ethanol by using 30, 40, 50, 60, 70, 80 and 90%, respectively. The solid–liquid ratio used in the extraction process was determined using ratios of 1:10, 1:15, 1:20, 1:30, 1:40, 1:50 and 1:60 (g/mL), and for the ultrasonic temperature determination, we used 20, 30, 40, 50, 60, 70 and 80 °C, respectively.

#### 3.2.4. The Orthogonal Optimization Test

An orthogonal test can be used analyze experimental results using fewer experimental groups to obtain better process parameters, and this method was adapted for use to determine the effects of various extraction parameters on the extraction of *B. striata* sunscreen components. Therefore, based on the results of the single-factor experiments, an orthogonal experiment was designed to optimize the extraction process. When designing the orthogonal test, the UVB absorption rate was taken as the evaluation index, and the factors for the four parameters of the solid–liquid ratio (A), ultrasonic temperature (B), ethanol concentration (C) and ultrasonic time (D) were taken as the independent variables. A 4-factor, 3-level L9 (3^4^) standard orthogonal table was selected to optimize the conditions for the extraction process of the sunscreen ingredients from *B. striata* in the UVB region. The individual test factors and levels used in this study are given in Table 7.

#### 3.2.5. UPLC-Q-TOF-MS Analysis

##### Sample Preparation for UPLC-Q-TOF-MS

The ethanol-extracted samples obtained under the optimized processing conditions from *B. striata* were filtered using 0.22 μm filter membranes.

##### UPLC-Q-TOF-MS Analysis Conditions

The chromatographic separation was performed using a Luna Omega Polar C18 column with 3 μm, 2.1 mm × 100 mm. The composition of the two mobile phases was 0.1% (*v*/*v*) formic acid in water (A) and acetonitrile (B): 0~6 min, 3%~20% B; 6~13 min, 20~45% B; 13~16 min, 45~75% B; 16~18 min, 75~95% B; 18~20 min, 95% B. The separations were performed with a constant flow rate of 300 μL/min. The column temperatures were set to 45 °C. Then, 5 μL of the samples were injected in full loop injection mode. We then performed electrospray MS detection in the negative detection mode with the ion source voltage set to −4.5 kV. Nitrogen was used as the sheath gas. The mass spectrum scanning range was set to 100~1500 Da. An 80 V declustering potential, −40 V collision energy and 10 V collision energy spread were used in the mass detection.

#### 3.2.6. Evaluation of the Sunscreen Effects of the Extracts from *B. striata* in the UVB Region

Preparation formulae for the sunscreen samples: Component A (glycerin 3.5% and Tween 80 0.72%, residual purified water); component B (stearic acid 3%, octadecyl alcohol 1%, liquid paraffin 11%, Span 60 5%, polydimethylsiloxane 2%, vitamin E 0.6% and sodium sorbate 0.3%); component C (carbomer 0.35%, EDTA disodium 0.05% and the appropriate amount of purified water); component D (triethanolamine 0.7%); Component E (*B. striata* extract 5~10%, appropriate amount of essence).

Preparation process: The fully swollen component C consisting of the water-soluble polymers at room temperature was heated to 75 °C and mixed. Components A and B were dissolved uniformly at 75~80 °C, and components B and C were slowly added to A while stirring them at 80 °C for about 15 minutes to obtain a uniform mixture. This was cooled to 50 °C, components D and E were added and the mixture was stirred. The temperature was reduced to 45 °C, and this yielded the sunscreen products. A control group sample (without *B. striata* extract), sunscreen Sample 1 (with 5% *B. striata* extract) and sunscreen Sample 2 (with 10% *B. striata* extract) were also prepared using the process described above.

The control and sunscreen Samples 1 and 2 prepared as described above were evaluated for their sunscreen effects using the national standard QB/T 2410-1998 [26]. This employed the 3M tape method, which is based on the principle that the UV-shielding agent and the UV absorbance in sunscreen cosmetics can block and absorb the UV rays from the sun. The samples were directly and evenly smeared onto the cuticle of the skin and other biological materials (such as a quartz pool and medical breathable tapes). This simulated the process of a person applying the cosmetic products onto the surface of their skin. A total of 8 mg of the products was pasted evenly onto a 1 × 4 cm piece of 3M medical tape on the transparent side of the quartz absorption cell and left for 30 min. A UV spectrophotometer was then used to measure the UV absorbance A value of the sample in the UVB region (280~320 nm), and the results of the sunscreen effect evaluation are shown in Table 6.

#### 3.2.7. Statistical Analysis

Statistical analysis was performed by using the SPSSAU website (https://spssau.com/index.html, accessed on 19 May 2024). The *t* test was used for the single-factor analysis, and the range analysis method was used to analyze the orthogonal experiment results. Statistically significant differences were labeled as * and ** for *p* < 0.05 and <0.01, respectively. GraphPad 8.0.2 was used to depict the graphs. PeakView 2.0 was used to load the results of the published compounds into the Analyst@TF 1.6 Software (PeakView 2.0) function block for analysis.

## 4. Conclusions

In this study, the UV absorption rate of the extracted chemical components of the orchid plant, *B. striata*, was determined. The orthogonal method was used to optimize the extraction process. The optimal extraction process was found to be when the ratio of solid-liquid was 1:40 (g/mL), the ultrasonic temperature was 60 °C, the ethanol concentration was 50% and the ultrasonic time was 50 min. Under these conditions, the extracts obtained from *B. striata* had a good absorption effect with respect to UVB, with an average UVB absorption rate of 84.38% and an RSD of 0.11%. This indicated that the process was stable and feasible. We subsequently used UPLC-Q-TOF-MS to identify eighteen compounds with sunscreen properties in ethanol extracts of *B. striata*, including eleven 2-isobutyl malic acid glucose oxybenzyl esters, four phenanthrenes, two bibenzyl and one α-isobutylmalic acid. These compounds are the basis for its sunscreen properties. The 3M tape method showed that the average Ai values of the sunscreen samples containing high and low concentrations of *B. striata* extracts in the UVB region were 1.201 and 0.727, respectively. This verified that the extracts from *B. striata* have a good UV absorption ability in the UVB region and they can achieve a sun protection effect in moderate sunlight. Therefore, the *B. striata* plant can be used as one of the candidates for cosmetic sunscreen purposes, and this study provides an experimental basis for the further development and utilization of *B. striata* in skincare products.

## Figures and Tables

**Table 1:** An L9 (3^4^) table of the sunscreen ingredient extraction parameters.

Experiment No.	Factors				Average Absorption of UVB (%)
	A: Solid–Liquid Ratio (g/mL)	B: Ultrasonic Temperature (℃)	C: Ethanol Concentration (%)	D: Ultrasound Time (min)	
1	1:30	40	50	40	79.74
2	1:30	50	60	50	78.70
3	1:30	60	70	60	78.48
4	1:40	40	60	60	79.02
5	1:40	50	70	40	76.30
6	1:40	60	50	50	84.34
7	1:50	40	70	50	75.78
8	1:50	50	50	60	78.56
9	1:50	60	60	40	80.72

**Table 2:** The results obtained for the range analysis of orthogonal experiments.

Item	Level	A: Solid–Liquid Ratio (g/mL)	B: Ultrasonic Temperature (°C)	C: Ethanol Concentration (%)	D: Ultrasound Time (min)
K value	1	236.92	234.54	242.64	236.76
	2	239.66	233.56	230.56	238.82
	3	235.06	243.54	238.44	236.06
K avg value	1	78.97	78.18	80.88	78.92
	2	79.89	77.85	76.85	79.61
	3	78.35	81.18	79.48	78.69
Optimum level		2	3	1	2
R		1.53	3.33	4.03	0.92
Number of levels		3	3	3	3
Number of repetitions per level r		3.0	3.0	3.0	3.0

**Table 3:** The results of the verification tests (*n* = 3).

Test Number	Weight of Medicinal Materials (g)	Average Absorption of UVB (%)	x ± *s* (%)
1	1.0002	84.40	84.38 ± 0.07
2	1.0001	84.28	
3	1.0000	84.46	

**Table 7:** The factors and levels of extraction parameters used for the orthogonal tests.

Level	A: Solid–Liquid Ratio (g/mL)	B: Ultrasonic Temperature (°C)	C: Ethanol Concentration (%)	D: Ultrasonic Time (min)
1	1:30	40	50	40
2	1:40	50	60	50
3	1:50	60	70	60

## Data Availability

The authors declare that all relevant data supporting the results of this study are available within the article.
